# Non-pharmacological Management in Palliative Care for Patients With Advanced COPD

**DOI:** 10.3389/fcvm.2022.907664

**Published:** 2022-07-18

**Authors:** Anna Pyszora, Agnieszka Lewko

**Affiliations:** ^1^Palliative Care Department, Collegium Medicum in Bydgoszcz, Nicolaus Copernicus University, Torun, Poland; ^2^Faculty of Health and Life Sciences, Coventry University, Coventry, United Kingdom

**Keywords:** palliative care, non-pharmacological management, COPD, narrative review, physiotherapy

## Abstract

Chronic obstructive pulmonary disease (COPD) is a disabling condition associated with progressive airflow limitation and lung tissue damage; its main symptoms are breathlessness, fatigue, cough, and sputum production. In the advanced stage of the disease, these symptoms may severely impact on a person's physical and psychological functioning, with some also developing chronic respiratory failure, associated with blood gas abnormalities. Non-pharmacological interventions can improve quality of life and functioning in the management of people living with advanced COPD. This article will provide an overview of common non-pharmacological methods used in the symptomatic management of severe COPD, including: breathlessness and fatigue management strategies, anxiety management, pulmonary rehabilitation (PR) and physical activity (PA), neuromuscular electrical stimulation (NMES), airway clearance techniques (ACTs), nutrition and non-invasive ventilation (NIV). The importance of a holistic and multi-disciplinary approach to people living with COPD will be discussed.

## Introduction

Chronic obstructive pulmonary disease (COPD) is one of the leading causes of chronic morbidity and mortality worldwide (1). COPD leads to mucous hypersecretion (chronic bronchitis), tissue destruction (emphysema) and small airway chronic inflammation and fibrosis (bronchiolitis) as well as systemic inflammation (2, 3). The progressive nature of the disease leads to severe poorly reversible airflow obstruction despite optimal bronchodilation therapy. This results in increased airway resistance and compliance and in consequence to air trapping, hyperinflation and flattening of the diaphragm (2, 3). The changes in mechanics of breathing in COPD lead to increased effort of breathing and energy expenditure at rest (4, 5). Further consequence of advanced COPD may be gas exchange abnormalities causing chronic hypoxaemia or nocturnal hypercapnia. The impact of systemic inflammation on other systems in the body are becoming more evident in advance stages of COPD. These consequences may include cachexia, skeletal muscle atrophy, osteoporosis, increased risk of cardiovascular disease or neuropsychiatric disorders (3). With the progression of the disease, the more frequent and more severe acute exacerbations lead to an increased risk of hospitalisations and deterioration of the function (6).

Consequently, COPD causes persistent and progressive respiratory and non-respiratory disabling symptoms, such as breathlessness, fatigue, cough, and/or sputum production (7, 8). It is also common for people with COPD to experience anxiety and depression (9). These symptoms negatively affect individuals with COPD including their health-related quality of life, activities of daily living, physical activity, and sleep (10). It is crucial to evaluate not only the intensity of these symptoms but also their impact on daily functioning and participation in family and social life. Importantly, patients with COPD had a 2-fold increased risk of frailty (11–13), which can affect prognosis and management in advanced COPD. Therefore, people suffering from severe COPD require a holistic and multi-disciplinary approach that involves a variety of healthcare professionals, including physicians, nurses, physiotherapists, occupational therapists, psychologists, and social workers (14). Palliative care is a multidisciplinary approach which focuses on patient's symptom management and improvement in quality of life, therefore it has to be incorporated earlier into the management of COPD (15).

This article provides an overview of methods used in palliative care for the non-pharmacological management of symptomatic patients with severe COPD, including the management of breathlessness, fatigue and anxiety, pulmonary rehabilitation (PR), neuromuscular electrical stimulation (NMES), management of sputum clearance, nutrition and chronic respiratory failure (e.g., non-invasive ventilation).

## Breathlessness Management Model in Palliative Care

**Figure 1:**
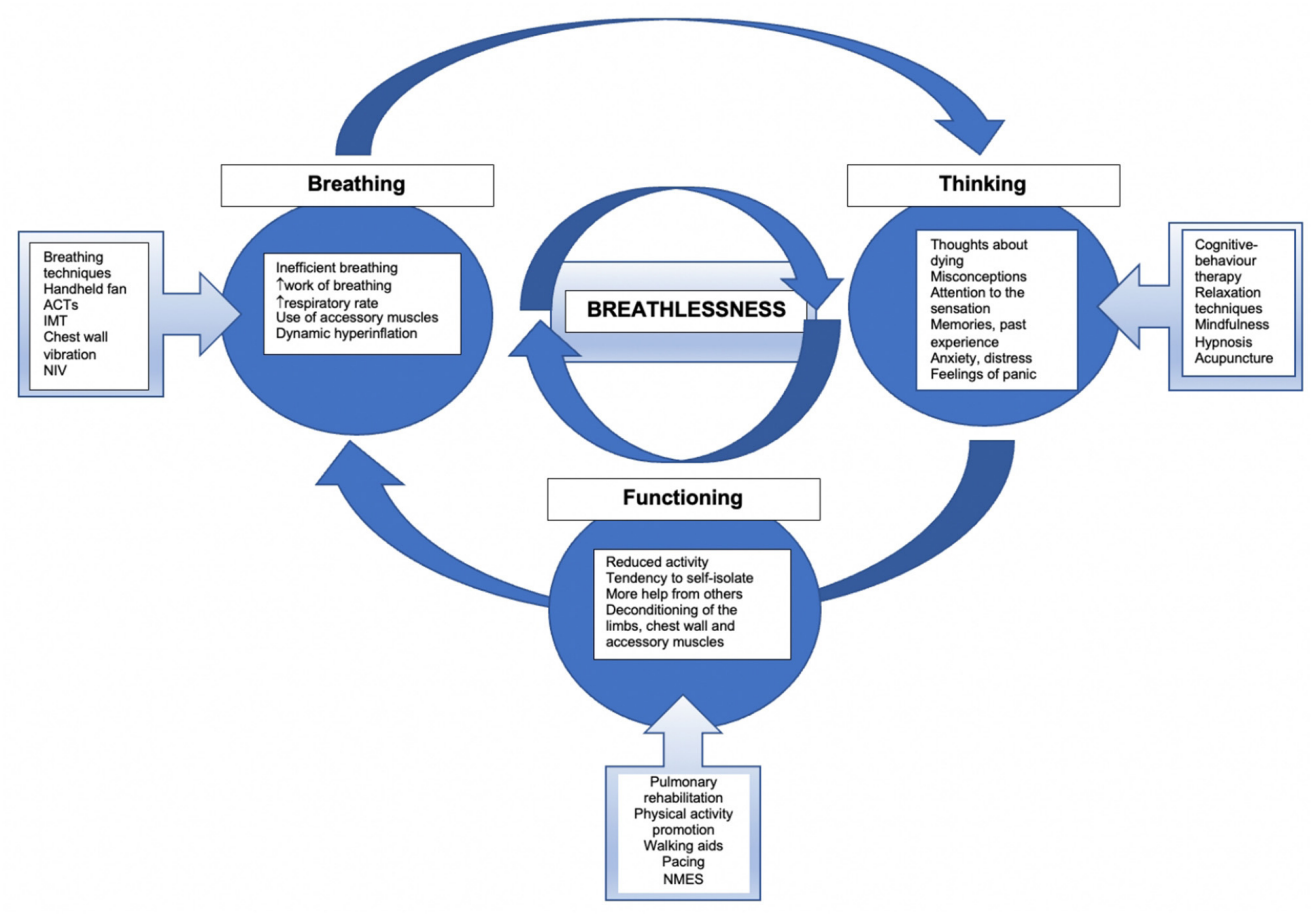
The model of breathlessness and management approaches. Adopted from Spathis et al. (21). ACTs, airway clearance techniques; IMT, inspiratory muscle training; NIV, non-invasive ventilation; NMES, neuromuscular electrical stimulation.

Breathlessness is the most common symptom in severe COPD (16), its prevalence is greater in the end stage of COPD (17–19). There may be several factors that contribute to the sensation of breathlessness (20). Management of this symptom should be multifactorial, based on an assessment of the patient to identify any elements contributing to the subjective sensation of breathlessness. The ‘Breathing, Thinking, Functioning’ model used by the Cambridge Breathlessness Intervention Service (CBIS), has been developed by Spathis and colleagues (21, 22). The model presents three features of the vicious cycle of breathlessness: (1) inefficient breathing, (2) thinking (including anxiety and distress), and (3) reduced function leading to muscle deconditioning (22, 23). With this model, it is possible to create categories of interventions to reduce breathlessness (see Figure 1) (21). Additionally, this model emphasized the need to implement multifactorial strategies to manage breathlessness.

## Day-to-Day Breathlessness Management Strategies

Breathlessness progresses with time, may intensify with advancement of the disease, and often negatively impacts on function (24). Individuals with advanced COPD will experience breathlessness on a regular basis. Their breathlessness may be triggered by exertion, for example during activities of daily living or a change in emotional state. Strategies to manage an acute onset of breathlessness may include positioning, breathing techniques, panic management, and desensitization (20).

### Positioning to Relieve Breathlessness

The “leaning forward” position is frequently used in clinical practice for the management of breathlessness triggered by activities of daily living or during rehabilitation. The theory behind this technique proposes that fixing the shoulder girdle, reduces activity of both the scalenes and sternomastoid muscles whilst increasing both transdiaphragmatic pressure (via diaphragmatic recruitment) and thoraco-abdominal movements (25–29). Using the forward lean to improve efficiency of respiratory muscles and decrease work of breathing is thought to lead to quicker recovery from breathlessness.

### Breathing Techniques

Although evidence supporting the effectiveness of breathing techniques varies, depending on the specific technique in question (30), their use is recommended to help breathless people gain better control of their breathing (31). Purse-lip breathing (PLB) technique has one of the strongest evidence-bases to support its use. The technique requires to inhale slowly through the nose and exhale through the mouth with the puckered lips, which alters respiratory mechanics (32). The increased resistance from half-opened lips on expiration physiologically generates an extrinsic positive end expiratory pressure (extrinsic PEEP), which decreases airway collapse by reducing the Bernoulli effect (33). This leads to decreased “air trapping” in patients with emphysema resulting in a reduction of hyperinflation.

Applying PLB lowers oxygen consumption, respiratory rate (RR) and reduces breathlessness in people with COPD (34). It shows to improve inspiratory capacity (IC) at rest (35) and reduced level dynamic hyperinflation during activity (36). PLB used on exercise shows to improve exercise tolerance (33, 37), reduce respiratory rate (RR) and improves recovery time in people with COPD (38).

Other breathing techniques, such as Breathing Control (BC), Blow as you go (BAYG) or Paced breathing, have less evidence to support their effectiveness, but some patients find them helpful in managing their breathlessness either on exercise or during recovery (39).

However, not all breathing techniques are beneficial for the management of breathlessness in severe COPD; diaphragmatic breathing may increase dyspnoea (40) by increasing chest wall asynchronicity (41, 42), leading to increase work of breathing. Another technique, slow deep breathing may predispose the diaphragm to fatigue (43). Therefore, clinical guidelines do not recommend use of either of these techniques for patients with advanced COPD (31).

### Walking Aid

Walking aids may be used to help with management of breathlessness during ambulation and enable patients with severe COPD to stay active and independent. The rollator frames on exertion may reduce work of breathing by maintaining the lean forward position during activity (44). Evidence from a research study by Probst at al. (45) shows rollator frames can significantly increase walking distance, whilst reducing exertional dyspnoea in patients with COPD. The effect on respiratory function was demonstrated with improvements in oxygen uptake, tidal volume and minute ventilation. Similarly, the use of gutter frames with elderly COPD patients have been shown to increase walking distance and reduce oxygen desaturation during ambulation (46).

### Handheld Fan

The benefits of utilizing cool, flowing air on the facial skin for patients with COPD is has long been known, with many patients reporting having benefited (47). The mechanism of action is explained in part through stimulation of facial temperature receptors (48) and modulation of central perception of breathlessness (49). Although a systematic literature review from 2008 showed insufficient data to support the evidence for fans' effectiveness (50), the authors emphasized that more research is necessary on selecting and identifying those who might benefit from using handheld fans (51, 52). Subsequently a number of studies showed that a cool draft of air from a hand-held fan directed to the face can be helpful in reducing the sensation of breathlessness in patients with advanced COPD (53–55). Moreover, there is data that suggests that using a handheld fan increases physical activity (56). Some authors indicate that future research should explore the relationship between handheld fan characteristics and relief of breathlessness (57). The authors researching handheld fans for breathlessness emphasize their acceptability to patients, relative inexpense, portability and ability to give patients more control; and recommended their use as part of palliative management to support patients' self-management and independence (53, 54, 58, 59).

## Long-Term Breathlessness Management Strategies

There are also some strategies to improve chronic breathlessness in the longer term. This includes exercise training or more comprehensive programmes such as pulmonary rehabilitation (PR) (60). Furthermore, in patients with COPD with dysfunctional breathing patterns, breathing retraining programmes may be considered. However, a systematic review by Holland et al. (30) demonstrated inconsistent evidence about improvement in breathlessness.

There is also evidence that Inspiratory Muscle Training (IMT) in moderate-to-severe COPD improves dyspnoea and quality of life (61, 62). A recent systematic review (63) presented results from 23 studies, which all indicated that IMT training decreased dyspnoea. However, there were some indications that improvement was limited to patients with pre-existing respiratory muscle weakness.

## Fatigue Management Strategies

The sensation of fatigue may be defined in various ways, including as tiredness (64), a lack of energy (65), exhaustion or weakness (2). The mechanism of subjective fatigue in COPD is complex and multidimensional (66, 67).

Exercise training alone or as a part of PR has been found beneficial in managing fatigue. A recent literature review demonstrated that any type of exercise could reduce fatigue (68). It has been also established that pulmonary rehabilitation reduces fatigue (60, 65), in particularly general, physical and reduce motivation (69).

One study investigated the effect of an 8-week progressive muscle relaxation programme on fatigue (70). It showed reduced fatigue and an improvement in sleep quality following the programme. There are some indications that sleep quality influences fatigue (71). Other fatigue management strategies reported by a qualitative study included pacing, protection, energy conservation, keeping active, resting or planning daily living and prioritizing (71, 72).

Energy conservation involves modifying an activity or the environment to decrease the level of energy required to complete a task. Pacing and energy conservation are also used for management of fatigue (73). A recent randomized controlled trial of a 2-week training programme involving energy conservation techniques (ECT) for COPD patients (74), demonstrated that after the programme there was lower level of desaturation and decrease in the metabolic equivalent of task (MET) while performing activities of daily living. Another observational study showed that ECT decreased heart rate, oxygen uptake, minute ventilation and dyspnoea. Although, ECT are recommended for management of fatigue in clinical practice, there is no evidence to demonstrate decrease of fatigue with this intervention.

## Anxiety Management

Feelings of anxiety are common in patients with advanced COPD (9). More intense breathlessness is associated with greater levels of anxiety (20). These symptoms negatively affect patients' physical functioning and increase their social isolation (75–78). Currently, we observe a growing number of studies addressing the issue of the use of non-pharmacological methods in the treatment of COPD patients affected by anxiety. The evaluated interventions included: relaxation (79, 80), hypnosis (81), cognitive behavioral therapy (82), and mindfulness (83). One study investigated also breathing techniques and found out them beneficial in managing anxiety (84). A recent systematic review, demonstrated significant, clinically relevant improvement in anxiety and depression following PR programme (85). Further research is needed to determine which interventions are the most effective and could be an efficacious add-on to standard PR programs or stand-alone treatment.

## Pulmonary Rehabilitation and Physical Activity

Pulmonary Rehabilitation (PR) is an important part of integrated care for patients with COPD. PR is a comprehensive programme which includes a variety of non-pharmacological interventions, exercise training and education (86). It has proved to be highly effective in management symptoms, improving QoL, physical and psychological functioning of patients with COPD (60, 85). In advance disease, people with COPD may experience frequent exacerbations and are at greater risk of frailty. Patients who complete PR after acute exacerbation would also have a lower risk of hospital readmission and mortality (87). Furthermore, COPD patients with frailty and risk of frailty showed benefit from PR, but they are more likely not to complete the programme (88).

Despite undisputable benefits from PR, in advanced stage of the disease, there may be number of barriers which could make attending programme difficult. The recent clinical report indicated that patients may struggle to complete post-exacerbation PR due to transport issues, advance disease, co-morbidities such as anxiety, poor motivation, and high fatigue (89). These patients are often fragile and may not always have the sufficient reserve to initiate the programme. It may be difficult for these patients to spend time outside home in late stage of disease, which may require effort and may create additional stress.

Nevertheless, the interventions aiming to reducing sedentary lifestyle and increase physical activity (PA) should still be considered. The evidence for different exercise-based PA-enhancing interventions is inconsistent. Behavioral change using tele-coaching or coaching, pedometers, applications, walking and home exercise programmes has been suggested to boost PA in patients with COPD (90, 91).

## Neuromuscular Electrical Stimulation

Impaired muscle function and decreased cross-sectional muscle mass are common features in people with severe COPD, which affect the respiratory and the skeletal muscles, especially of the lower limbs (92, 93). Patients with advanced COPD who are severely affected by muscle weakness, including those who are housebound, may benefit from Neuromuscular Electrical Stimulation (NMES) (94). NMES usually is applied to the quadriceps muscle and improves impaired muscle function and structure by increasing cross-sectional muscle mass, muscle force, endurance, and exercise tolerance as well as reducing dyspnoea (94–96). Moreover, some studies report that NMES promotes a reduction of the perceived sensation of dyspnea during exercise in patients with COPD (97). For people admitted to an intensive care or high dependency unit with an acute exacerbation of COPD, research suggests NMES combined with conventional exercise may reduce the time taken for patients to first sit out of bed (98).

The effectiveness of NMES in adults with advanced COPD and other diseases was analyzed in two Cochrane Systematic Reviews (50, 98). The authors conclude that there is a high strength of evidence that NMES may be an effective treatment for muscle weakness in adults with advanced progressive disease.

## Airway Clearance Management

For some COPD patients, cough and sputum may be a burden, especially during exacerbations. When the patient experiences difficulties with sputum expectoration, advice and support may be required. There are several airway clearance techniques (ACTs) recognized as effective methods (99, 100). Application of ACTs decreases breathlessness, lower need for ventilatory assistance and Positive Expiratory Pressure (PEP) devices improve sputum volume expectoration and decrease hospital length of stay for COPD patients admitted due to acute exacerbation (101, 102). In a recent review, significant improvements in the rate of exacerbation frequency at 6 months of ACTs use was demonstrated (100). Therefore, it would be important to review if sputum is cleared effectively and identify potential need for management with appropriate ACTs in COPD patients.

## Nutrition Management

Many people in advanced stages of COPD are underweight and may demonstrate sign of cachexia. Evidence suggests that 25–40% of all COPD patients have low body weight, 25% of patients have moderate to severe weight loss, and 35% of patients with extremely low fat-free mass (FFM) index (103). This has a negative effect on muscle mass and function and impacts exercise tolerance. Therefore, the European Respiratory Society (ERS) recommends that nutritional interventions should be considered as a single treatment or integrated with exercise training (104). Especially, patients with negative energy balance, may benefit from energy- and protein-enriched diet and the evidence suggests that nutritional supplementation promotes weight gain among patients with COPD (105). Furthermore, because exercise increases energy expenditure, it is suggested to assess the nutritional status of COPD patients before starting Pulmonary Rehabilitation (86). In patients with weight abnormalities, dietary counseling and food fortification or nutritional supplementation should be considered. Some authors suggest that smaller volumes of food may be more appropriate to optimize energy intake (106). Education and advice on nutrition are indicated as methods that bring a short-term effect on improving intake (106).

## Ventilatory Support

**Figure 2:**
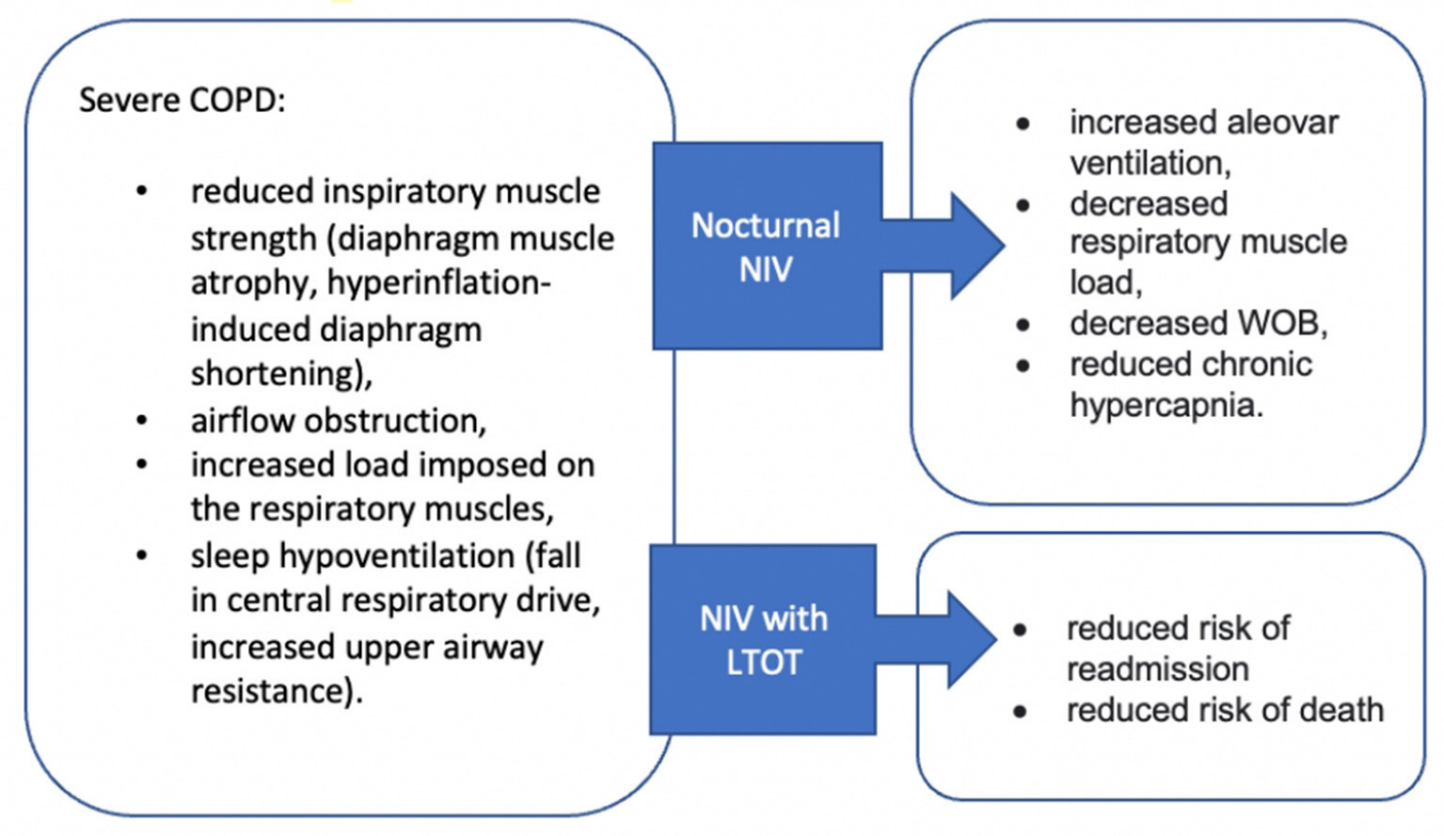
Effectiveness of Non-invasive ventilation in patients with advanced COPD. NIV, non-invasive ventilation; WOB, work of breathing.

Due to the small airway disease and lung hyperinflation, the diaphragm muscle is flattened, which may lead to its atrophy and greater fatiguability in severe COPD (107). Sleep hypoventilation is also observed in some people in advanced COPD (108). These factors may lead to a development of the type 2 respiratory failure. Therefore, these patients may benefit from nocturnal non-invasive ventilation (NIV) to support their respiratory muscle. The American Thoracic Society (ATS) Clinical Practice Guidelines recommend the use of nocturnal NIV in addition to usual care for patients with chronic stable hypercapnic COPD (109). A systematic review on the use of NIV in severe stable COPD concluded that bilevel non-invasive positive pressure ventilation may have an adjunctive role in the management of chronic respiratory failure through attenuation of compromised respiratory function and improvement in health-related outcomes (110). There is also evidence that long-term NIV added to home oxygen therapy reduces risk of readmission and death (111). Figure 2 highlights the key benefits from NIV. Furthermore, it is important to consider the application of appropriate therapeutic pressures, which is the key factor guaranteeing clinical effectiveness for carbon dioxide level reduction (112). However, McEvoy et al. (113) emphasizes that whilst nocturnal NIV in stable oxygen-dependent patients with hypercapnic COPD may improve survival, this appears to be at the cost of worsening quality of life. Hence, it is important to take into consideration individual patient preferences and agree on the treatment plan collaboratively.

This article discussed several non-pharmacological interventions used to manage symptoms and clinical problems arising in the palliative care for patients with advanced COPD. The summary of these various interventions, their evidence and clinical practice recommendations are presented in Table 1.

**Table 1:** Effectiveness of non-pharmacological interventions used in palliative care in COPD.

**Intervention**	**Help to manage**	**Stength of evidence**	**Clinical practice recommendations**
Pulmonary Rehabilitation	Breathlessness, fatigue, anxiety, improves exercise tolerance	+++ (60, 87, 114–116)	BTS/ACPRC guideline (31) ERS/ATS statement (86)
Positioning to relieve breathlessness	Breathlessness, anxiety	x	BTS/ACPRC guideline (31)
Breathing techniques	Breathlessness, anxiety	+++ PLB-Pursed-lip breathing (117, 118) x (BAYG- blow as you go, BC- breathing control, PC-paced breathing)	BTS/ACPRC guideline (31) Diaphragmatic breathing not recommended by BTS/ACPRC guidelines (31)
Respiratory muscle training	Breathlessness, Fatigue	+++ (119)	BTS/ACPRC guideline (31)
Breathing retraining	Breathlessness	++ (50)	-
Walking aid	Breathlessness	++ (45, 50)	Rollator frame and a gutter rollator frame are recommended by BTS/ACPRC guideline (31)
Handheld fan	breathlessness	+++ (52, 53, 55, 56)	-
Chest wall vibration	breathlessness	+++ (50)	May be difficult to use in practice.
Energy conservation techniques	fatigue	+ (74)	BTS/ACPRC guideline (31)
Airway clearance techniques	Breathlessness	-	ACBT, AD, OPEP are recommended (31, 120)
Relaxation	Breathlessness, Fatigue, Anxiety	x (79, 80)	BTS/ACPRC guideline (31)
Non-invasive ventilation	Breathlessness, Fatigue	+++ (110, 113)	BTS/ACPRC guideline (31) ATS clinical practice guideline (109)
Neuromuscular Electrical Stimulation	Breathlessness	+++ (50, 96, 121) x (95, 97)	(120)
Acupuncture		++ (50)	-

*+++ Strong evidence (based meta-analysis, systematic reviews); ++ Moderate evidence (based on few RCT); + weak evidence (based on non-randomized studies); x not sufficient evidence to support effectiveness, BTS, British Thoracic Society; ATS, American Thoracic Society; ERS, European Respiratory Society; ACBT, Active Cycle of Breathing Techniques; AD, Autogenic Drainage; OPEP, Oscillatory Positive Expiratory Breathing*.

## Conclusion

For patients in the advance stage of COPD, whilst a ceiling effect in pharmacological treatment is often reached, there is a range of management strategies which could be used to improve their quality of life, as it was presented it this article. There are several interventions suggested for relief of symptoms in clinical practice, but not all the methods have a strong evidence-base to support their effectiveness. However, palliative care does not always fit the Evidence-Based Medicine framework (122). Whereas breathlessness received the greatest attention and there is a wide body of evidence to support management of this symptom. Hence, there are specially designed services to address this problem. Other symptoms, such as fatigue, may be acknowledged, but there are not always specifically treated or may lack the complex management approach. This is potentially the reason why, the palliative care for COPD patients is often fragmented and interdisciplinary approach not always well-coordinated. The palliative care for patients with COPD should be a key part of the long-term management plan and a gold standard of care in advanced COPD. Therefore, there is a need for more research into management of symptoms other than breathlessness and development of more complex management programmes for palliative management in COPD.

## Author Contributions

Both authors listed have made a substantial, direct, and intellectual contribution to the work and approved it for publication.

## Conflict of Interest

The authors declare that the research was conducted in the absence of any commercial or financial relationships that could be construed as a potential conflict of interest.

## Publisher's Note

All claims expressed in this article are solely those of the authors and do not necessarily represent those of their affiliated organizations, or those of the publisher, the editors and the reviewers. Any product that may be evaluated in this article, or claim that may be made by its manufacturer, is not guaranteed or endorsed by the publisher.
